# The Whitney Case; with Prof. Bennett’s Letter

**Published:** 1859-09

**Authors:** 


### THE WHITNEY CASE.

"We, the Academy of Medicine, after a full examination of the reports of the case, and the post mortem examination, do consider that the death of Mr. Whitney was in nowise the consequence of improper treatment, but was the unavoidable result of *a complication of disorders.*"

(*Proc. Acad. Med., N. Y.*)

It is much to be regretted that the Academy of Medicine, in delivering their opinion in reference to the above case, did not think proper to enter more into detail and state the grounds of that opinion. We once knew an indifferently-educated member of our profession, when interrogated rather closely as to the nature of a disease, take refuge in the same vague expression, and say his patient had "a complication of disorders."

The simplest form of disease seems often obscure enough, but when told it results from a complication of disorders, none but active and enquiring minds (and such are not those of the majority) will choose to trouble themselves with what is mystical and unintelligible.

When we consider how excited the public have been in connection with this case, we would expect the Academy to feel a responsibility rested on them to decide less indeterminately the matter in question.

As their "resolution" leaves it, it not only does not award a full measure of justice, but gives no satisfaction whatever to the medical men more particularly concerned or to the public. The omission, however, has been supplied elsewhere. Professor Bennett, of Edinburgh, and the celebrated Rokitansky, of Vienna, have not hesitated to state their views. We publish Professor Bennett's letter, and refer our readers to the columns of the *American Medical Monthly*, for September, where they will find Rokitansky's communication.

We have hitherto abstained from all expression of opinion as to the relations that have arisen to the parties so unfortunately identified with the case.

We do not impute to any, improper motives in introducing to the notice of the public a supposed disastrous result of proceedings, conceived and conducted, as we believe, in accordance with science. But we do deplore what must appear to many a want of professional decorum and a seeming disregard of another's feelings. Occurrences like these tend to lower the profession in public estimation, and to lessen confidence amongst those who, from community of pursuits, of sentiments, and, to some extent, of position, should be linked in sympathy together. It is a mistake also to suppose that he who disparages, will rise in proportion as he sinks another's reputation—quite the reverse. He who thus lowers his profession, equally with the rest, suffers in public esteem, participates in the degradation he has inflicted on another, and earns the distrust of his fellows, who well know that inferior minds are sometimes quick-sighted enough to detect errors, though perhaps unable to correct them.

---

### PROFESSOR BENNETT'S LETTER.

EDINBURGH, *May* 18, 1859.

MY DEAR DOCTOR—I have hesitated for some time as to how I ought to reply to your letter; that is, whether it would be better to enter into a lengthy criticism of Mr. Whitney's case, or simply answer the two queries you have put me. After carefully studying the case itself, I find so much to comment on in the various branches of diagnosis, pathology, treatment and ethics, that I feel constrained to throw my notes aside, and give up the idea of entering into the matter fully, as it would oblige me to write a treatise rather than a letter.

You request my opinion: As to the tubercular character of the pulmonary abscess; 2. As to whether such effect as Dr. Beales intimates could have been produced by the injection of nitrate of silver, which was employed.

1st. *As to the tubercular character of the pulmonary abscess.*—The description given by Dr. Green of the physical signs on the 25th October, 1858, is remarkably clear (see *Am. Monthly*, p. 104), and can leave no doubt as to the existence of condensation of the left lung at the apex, with softening of the tissue. When Dr. Beales was called in, Dec. 14th, he does not seem to have made any physical examination of the chest. At all events, no account of any is given from that time up to the period of the patient's death (*Monthly*, p. 111 to p. 115). But he tells us afterwards, that he had previously made such examination (p. 117) towards the end of October; he gives no description of these signs, but only states his opinion, viz., that "he could not discover any tubercles in his lungs, and did not believe any existed." The report of the post mortem, however, demonstrates that the physical examination of the lung made by Dr. Green was in every respect correct (*Monthly*, p. 116). "The whole of the upper part of this lobe (the left) was red, and solid—hepatized." "At the commencement of the bronchial ramifications there was an open cavity, about the size of a small black walnut, of a reddish-brown color, and irregular villous surface, as though a slough had separated." This answers thoroughly for the flat sound, detected two months previously, over the upper portion of the left lung, and the humid rale audible below the left clavicle, on inspiration and expiration."

As to the nature of the disease, Dr. Beales conceives it to have been acute and recent pneumonia (p. 118). I cannot think so, when I consider the accurate account of physical signs given two months previously, then indicating the condensation and softening which were subsequently found. It must have been chronic pneumonia passing into gangrene, or a limited tubercular abscess, accompanied by chronic pneumonia of the apex. The descriptions given of the lesions do not enable me to say which of these it was. In fact, I consider it a matter of little importance, because a chronic exudation of the apex so readily passes into tubercle, and an old tubercular abscess is so commonly accompanied by chronic pneumonia, that, in truth, it often becomes difficult, if not impossible, to say where tubercle ends and pneumonia begins. It is enough that a chronic *exudation* was there, and that, I consider, Dr. Green fully indicated by his physical signs six or seven weeks before Dr. Beales was called in.

2d. *As to whether the treatment caused the disease.*—Dr. Beales informs that Dr. Mott told the family, after the post mortem examination, that they had not seen any disease that might not have been produced within a week (*Monthly*, p. 118). According to this idea, it could not have been the injection into the trachea which caused the pulmonary disease, as that operation was performed on the 6th of December, without causing any irritation, and he died on the 21st. Besides, the pulmonary lesion existed on the 25th October, as proved by physical signs. The injection, therefore, could not have caused that lesion. Is it not more probable that, a chronic exudition having existed at the apex, and a cavity formed, this latter perforated the pleura, causing the pleural exudation, followed by the emphysema of the cellular tissue? At what moment did this perforation take place? We are told that Mr. Whitney visited Dr. Green on the 14th of December, after breakfast (the time is not stated); the doctor passed an instrument into his throat, and finding some obstruction, he pushed the instrument with some force; he (Mr. W.) felt something give way, immediately experienced *severe* pain about the top of the windpipe, and told the doctor he had "hurt him;" he returned home, informed the family of what had occurred, and was seen by Dr. Beales at 1 P. M. (p. 112). Dr. Green's account of what happened on the 14th is different. According to him (*Monthly*, p. 106), when the sponge reached the glottic opening, the patient partially closed the throat, so that the instrument did not enter the windpipe at all. It was at once removed, no more force having been used than that which is constantly employed every day in operations on the air-passages. The patient, after talking awhile with Dr. Foy and myself, and remarking that "the operation hurt him more," or that "he felt it more than usual," he left, with the arrangement that he should return the next day and have the tube employed. The patient's account to Dr. Beales is, that at the moment the probang was arrested at the glottis, he felt something give way, and experienced *severe* pain about the top of the windpipe (p. 112). But is this consistent with the fact that he talked for awhile with Drs. Foy and Green, and went home, intending to come next day, etc.? Again, was the pain, if felt *at the top* of the windpipe, symptomatic of perforation of the pleura in the chest? It appears to me difficult to answer these questions, more especially when we read the account of the alarming condition of Mr. W. at 1 o'clock, when first seen by Dr. Beales (*Monthly*, p. 111). I am, therefore, inclined to ask, What occurred in the interval between the patient's leaving Dr. Green and his seeing Dr. Beales? This may have been an interval of three or four hours, and of this most important period I can find no account whatever. It is true, Mr. Whitney seems to have had the impression that his severe symptoms commenced when the sponge was arrested, but this is negatived by the evidence of Drs. Green and Foy, who saw no evidence of severe pain, even in the throat. When, then, did the pulmonary and pleural perforation take place, which induced the emphysema and subsequent symptoms? With the facts at present before me, I cannot answer this question, but it is clear to my mind that it may have occurred during the hours not accounted for; that it might have been altogether spontaneous, connected only with the progress of the chronic pulmonary lesion, and that it had no relation whatever to the operation performed by Dr. Green.

In addition to the lesion already referred to, it appeared, on dissection, that there was an abscess behind the larynx, and stretching towards the left of the pharynx, the size of a hen's egg, with an opening at its upper and posterior part, into the pharynx, large enough to admit the end of the forefinger. This abscess was not discovered before death, by any of his attendants, medical or surgical. Dr. Mott, a great authority in surgery, asserts that this was an acute abscess. Dr. Beales distinctly states (*Monthly*, p. 119), that in his opinion it was caused on the 14th December, by the accidental laceration of the pharynx by the probang; and his account of the symptoms, as he observed them at 1 P. M. on that day, supports the supposition, especially the intense pain in the region of the larynx, shooting through to the cervical vertebræ, and down the course of the trachea to the chest; he kept grasping the larynx, etc., (p. 111). Now, as the larynx was shown subsequently to be healthy, it is very probable that these symptoms were connected with the pharyngeal abscess. But how? Is such pain consistent with the first commencement of an abscess, or did it accompany the rupture of an abscess previously formed? By what signs was it pronounced to be acute? Might it not have existed before the operation of the 14th? Might it not have come on subsequently? On all these points I will not venture to speak. But having now used the probang in many hundred cases, and under all circumstances, I am at a loss to understand how its being arrested at the glottis would either lacerate the pharynx, so as to give rise to an abscess, or cause the bursting of an abscess which had already formed—"downward, behind and below the thyroid cartilage." I cannot but think that the application of the sponge had no more to do with this abscess than it had with the exudation into the apex of the left lung.

Yours, very truly and sincerely,

J. HUGHES BENNETT.

